# Completeness and Audibility of Verbal Orders for Medications and Blood Products during Trauma Resuscitation

**DOI:** 10.5811/westjem.18585

**Published:** 2025-11-26

**Authors:** Rebecca Ryan, Kathleen Williams, Jamie Aranda, Nancy Jacobson

**Affiliations:** Medical College of Wisconsin, Department of Emergency Medicine, Milwaukee, Wisconsin

## Abstract

**Introduction:**

Resuscitation of critically injured patients requires effective team leadership. Poor communication is the leading cause of sentinel events. Closed-loop communication reduces error during trauma resuscitations. Nonetheless, previous studies show few verbal orders are audible. Verbal orders during trauma resuscitations have not been studied for completeness. In this project we aimed to assess whether verbal orders for medications and blood products during trauma resuscitations were complete, audible, and used closed-loop communication.

**Methods:**

This was an observational assessment of a convenience sample of verbal orders that trauma captains gave for medications and blood products during the primary and secondary survey. It was conducted in an academic emergency department (ED) at an adult Level 1 trauma center. We assessed medication orders for the presence or absence of medication name, dose, and route. Blood orders were evaluated for the presence or absence of blood product (packed cells or whole blood) and type (O− or O+). We recorded orders as audible or inaudible. Closed-loop communication was recorded as present or absent. Orders were considered complete if they included all elements. We used descriptive statistics to analyze data.

**Results:**

There were 186 verbal orders enrolled: 165 (88.7%) for medications and 21 (11.3% for blood products. For medication verbal orders, 77.9% (n=127) were audible, 73.6% (n=120) included the name, 62.0% (n=101) included the dose, 17.8% (n=29) included the route, and 73.5% (n=111) used closed-loop communication. Overall, 23 (14.1%) medication verbal orders were complete. Regarding verbal orders for blood, 16 (76.2%) were audible, three (14.3%) included the blood product, seven (33.3%) included the blood type, and 13 (61.9%) used closed-loop communication. Overall, 0% (n=0) of the blood product verbal orders were complete.

**Conclusion:**

Audible, complete verbal orders, and closed-loop communication were underused during trauma resuscitations. Interventions to improve communication of verbal orders warrant evaluation in the ED.

## INTRODUCTION

Resuscitation of critically injured patients requires effective communication. The Joint Commission names poor communication as the leading root cause of all sentinel events.^1^ In one survey-based study of healthcare professionals, 14% of respondents reported observing a medication error occurring due to misunderstanding or mishearing a verbal order.^2^ Closed-loop communication reduces errors during verbal ordering and decreases time to task completion.^1^,^3^ In a 2005 study of verbal orders during trauma resuscitations, only 16% of verbal orders were audible and only 6% were understandable.^4^ Despite studies indicating poor communication during trauma resuscitations, verbal orders during trauma resuscitations have not been studied for completeness.

Blood product verbal orders have become more complex in recent years due to the successful implementation of whole blood resuscitation algorithms in civilian trauma programs.^5^ Research supports the use of whole blood as the resuscitation product of choice in the treatment of hemorrhagic shock and has shown improved outcomes in military settings.^6^–^10^ In addition to whole blood products, more traditional blood products such as O- or O+ packed red blood cells, plasma, and platelets are often administered to traumatically injured patients. In this ED, trauma patients are given uncrossed packed red blood cells (O+ for male and O- for female) or low titer O+ whole blood for resuscitation of hemorrhagic shock in all male patients with anticipated activation of massive transfusion.

The use of multiple blood products (packed red blood cells and whole blood) and blood types (O+ and O−) introduces the potential for error wherein an incorrect blood product or blood type could be administered. Blood administration errors should be avoided in all patients but are particularly worrisome for patients with Rh-negative blood who may become pregnant. This is due to the potential for late complications such as hydrops fetalis, thrombocytopenia, neutropenia, and hemolytic disease of the fetus and newborn.^11^–^13^ A sentential event involving the administration of O+ blood to a Rh-negative female trauma patient was the catalyst for this study.

Medication verbal orders are similarly complex because resuscitation of critically ill trauma patients may require the use of numerous medications given via multiple routes and using different units. Tailoring doses of medications to physiologic parameters may be required, particularly for patients at extremes of age or with unstable vital signs. Simply put, a one-dose-fits-all strategy is inappropriate in this setting. Once a medication, dose, and route have been decided by the ordering physician, these must be specified clearly.^14^

Given the importance of clear communication during trauma resuscitation, our aim in this study was to assess medication and blood product verbal orders during trauma resuscitations in the ED for audibility, completeness, and closed-loop communication.

## METHODS

### Study Design

This was an observational study of a convenience sample of verbal orders for medications and blood products provided by the trauma captain or a supervising physician during the primary and secondary survey of trauma activations at this adult Level 1 academic trauma center. This study was reviewed by the institution’s institutional review board (IRB) and was determined to be IRB exempt.

### Study Setting

We conducted this study in an academic emergency department (ED), an adult Level 1 trauma center that sees approximately 4,000 trauma activations per year. Trauma activations are divided into trauma alerts (higher acuity requiring immediate life-saving intervention) and trauma calls (mid-acuity with high likelihood of requiring additional resources). Activation criteria for trauma alerts and trauma calls are included in Table 1. The primary and secondary surveys of trauma patients take place in one of four ED resuscitation bays and follow Advanced Trauma Life Support guidelines. During trauma activations, medications, blood, and interventions are ordered verbally by the trauma captain. Orders are not placed into the electronic health record (EHR) until after the secondary survey when the physician team can step away from the patient bedside.

**Population Health Research Capsule**

What do we already know about this issue?

*Resuscitation of critically injured patients requires effective communication, yet studies show few verbal orders are audible and completeness has not been studied.*

What was the research question?

*This project assesses verbal orders during traumas for completeness, audibility and closed-loop communication.*

What was the major finding of the study?

*Complete verbal orders were utilized 14.1% of the time for medications and 0% of the time for blood administration during traumas.*

How does this improve population health?

*This project demonstrates that interventions to improve communication of verbal orders warrant evaluation in resuscitation settings.*

**Figure 1:**
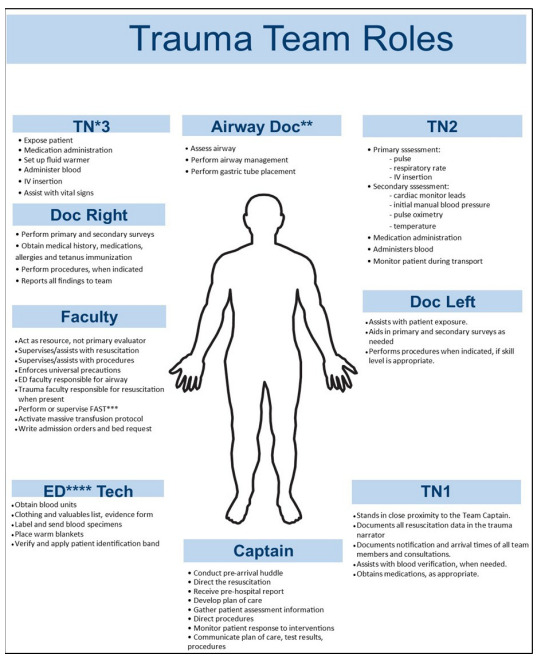
Trauma resuscitation team roles. PGY-3 EM residents in this three-year EM program and PGY-3–5 surgery residents serve as trauma captains with close faculty supervision. *DOC*, doctor (referring to a resident or attending physician); *ED*, emergency department; *FAST*, focused assessment with sonography in trauma; *PGY*, postgraduate year; *TN*, trauma nurse

Postgraduate year (PGY)-3 emergency medicine (EM) residents in this three-year EM program and PGY 3–5 surgery residents serve as trauma captains with close faculty supervision. In addition to the trauma captain, other team members present at trauma activations and team roles are shown in Figure 1. Roles include the following: resident physicians; trauma surgery and emergency medicine faculty; ED nurses; ED techs; ED pharmacists; respiratory therapists; and social workers. The specific personnel and resources mobilized for a trauma alert and a trauma call are detailed in Table 1.

The most notable difference is that a trauma surgery faculty responds immediately to any trauma alert, but within six hours to a trauma call. Team composition does not change with time of day or day of week. Each trauma activation is a single resuscitation. A single trauma team is not expected to care for more than one trauma resuscitation at a time. Resuscitations are run in the trauma bay, a large area with four separate resuscitation spaces separated by curtains. Anywhere from 0–4 patients are resuscitated in this space at the same time.

### Study Protocol

Trauma activations including both trauma alerts and trauma calls were observed by a member of the project team. We included a convenience sample of trauma activations, which were observed at all hours of the day and all days of the week over a 20-month period. Key measures were recorded on a password-protected tablet by a project team member in real time. The project team member collecting data was located near the recording nurse (TN1 [trauma nurse 1] in Figure 1), within five feet of the trauma captain, to ensure environmental noise did not impact data collection.

Data were collected by a medical student with prior experiences as an ED technician at a medium-sized urban ED, although without ATLS training. The data collector was not a member of the trauma team prior to or throughout the observation period. Given the dicohotomous nature of the variables observed, we decided that ATLS certficiation was not necessary to perform adequate data collection.

### Key Measures

Key measures were dichotomous variables including the presence or absence of the following: order audibility; medication name/dose/route; blood product and type; and closed-loop communication. Patient sex, acuity of trauma, and the training program of the captain were also recorded.

#### Order Audibility

Verbal orders were considered audible if the project member collecting data could hear the order being given. We classified orders as inaudible in the following circumstances: an order was called but was indiscernible; closed-loop communication was used but no initial order was heard, or when the ED pharmacist brought a medication to bedside following a verbal order that was not heard by the project team member. Because the project team member was closer to the captain than the bedside registered nurse (RN), the ED pharmacist, or the recording trauma RN, it is presumed that if the order was inaudible to our data collector, it was likely inaudible to other team members standing farther away.

#### Order Content

No clear standard or recommendation exists for contents of a verbal order in a resuscitation situation. Following the sentinel event that prompted this study, a multidisciplinary group including trauma surgery faculty, emergency medicine (EM) faculty, ED nursing, ED pharmacists, and a patient safety specialist determined expected verbal order contents during trauma resuscitations. As per the Institute for Safe Medication Practices, a complete medication order includes the drug name, dose/strength, frequency, route of administration, indication, type/frequency of assessment to monitor the effects of therapy, drug administration precautions, drugs to discontinue during therapy, and instructions to address known potential emergencies associated with the drug. Our approach to selecting the contents to define a complete verbal order was that a verbal order should contain the components suggested for a complete written order that are both relevant to the emergent bedside resuscitation of a critically injured patient and can be stated with a pace commensurate to the resuscitation itself. In this way, the content of medication verbal orders including name, dose, and route were selected by consensus of this multidisciplinary group and recorded as present or absent during data collection. If all three of these were present, the verbal order was considered complete (eg: “Give 100 mg of ketamine, IM” [intramuscular).

We did not include components such as frequency, monitoring effects of therapy, drug precautions, drugs to discontinue, or instructions to address potential emergencies. We decided that these components did not apply to a trauma resuscitation given that the physician and nursing team members are present at bedside continuously throughout the resuscitation, that the team is witnessing any effect of the medication in real time, and that the frequency is presumed to be “once, now” given the context of the verbal order. The content for blood verbal orders were selected based on input of our multidisciplinary trauma/EM team. Departmental recommendations were provided based on the availability of blood products in our ED and the necessary verbal components to distinguish between these blood products. The content of blood verbal orders including blood product (packed red blood cells or whole blood) and blood types (O+ or O− were recorded as present or absent). A blood order was considered complete if both component parts were present (eg: “Administer one unit of O negative packed red blood cells.”)

### Closed-loop Communication

We defined closed-loop communication as audible repetition of the verbal order and recorded as present or absent for all medication and blood orders. Repeat orders made by the trauma captain after another team member used closed-loop communication to clarify were not included in the study. We documented closed-loop communication for the original verbal order as being present.

### Data Analysis

Data were housed in a secure Excel spreadsheet (Microsoft Corporation, Redmond, WA). We used descriptive statistics to analyze all data. Order content was analyzed for completeness, with subgroup analysis of component parts. Additionally, we performed subgroup analyses based on the sex of the patient, the acuity of trauma, and the training program of the ordering physician. Specifically, we sub-analyzed high-acuity (trauma alerts) and mid-acuity (trauma calls due to differing degrees of critical injury contributing to different numbers of team members involved, different medication and blood products administered, and the level of urgency of resuscitation.

## RESULTS

There were 186 verbal orders enrolled over 23 months between February 2020–December 2021. Medication orders comprised 88.7% (n=165/186) of enrolled orders, and blood orders comprised the remaining 11.3% (n=21/186). Types of medications ordered and recorded included analgesics, anxiolytics/anti-psychotics, sedatives and paralytics for rapid sequence intubation, intravenous fluids, antibiotics, and tetanus vaccines. Of the trauma resuscitations, 38.7% (n=72/186) were trauma calls and 61.3% (n=114/186) were trauma alerts. Trauma captains gave 92.4% (n=172/186) of verbal orders, and the remaining 7.5% (n=14/186) were provided by another supervising physician. Of orders given by trauma captains, 80.2% (n=138/172) were given by an EM resident and 19.8% (n=34/172) were given by a surgery resident. We documented that 71.0% (n=132/186) of the verbal orders were given during resuscitation of male patients with the remaining 29.0% (n=54/186) of verbal orders during the resuscitation of female patients.

### Order Audibility

Of all verbal orders given for medications, 77.9% (n=127/163) were audible. Subgroup analysis based on acuity of trauma revealed that 77.9% (n=53/68) of orders given during trauma calls (mid-acuity with high likelihood of requiring additional resources) were audible, and 77.9% (n=74/95) of orders given during trauma alerts (higher acuity requiring immediate life-saving intervention) were audible. Subgroup analysis based on trauma captain residency program demonstrated that 80.2% (n=93/116) of orders given by an EM resident were audible, 67.6% (n=23/34) of orders given by a surgery resident were audible, and 80% (n=8/10) of orders given by a supervising physician were audible.

Similar to medication verbal orders, 76.2% (n=16/21) of orders for blood were audible. Subgroup analysis based on acuity of trauma revealed that 75% (n=3/4) of orders during trauma calls were audible and 76.5% (n=13/17) of orders during trauma alerts were audible. Subgroup analysis based on trauma captain residency program revealed that 75% (n=15/20) of orders given by an EM resident were audible and 100% (n=1/1) of orders given by a supervising physician were audible. No verbal orders for blood were given by a surgery resident.

### Order Content

Only 14.1% (n=23/163) of medication verbal orders were complete, with 73.6% (n=120/163) including the name, 62.0% (n=101/163) including the dose, and 17.8% (n=29/163) including the route. Subgroup analysis based on acuity of trauma revealed that 14.7% (n=10/68) of medication verbal orders during trauma calls were complete, and 13.7% (n=13/95) of medication verbal orders during trauma alerts were complete. Subgroup analysis based on trauma captain residency program revealed 17.2% (n=20/116) of medication verbal orders given by an EM resident were complete, 8.8% (n=3/34) of orders given by a surgery resident were complete, and 0% (n=0/10) given by a supervising physician were complete.

Throughout the course of data collection, we noted that the unit of a dose of medication was frequently not included in the verbal order. Unit was not initially included in the data collection template, although after recognizing the frequent absence of the unit in the medication verbal order, the presence or absence of a unit was subsequently recorded. There were 100 medication verbal orders where unit was included in our assessment. Of these 100 verbal orders, it is evident that similar to other parts of the verbal order, unit was underused, while 4% (n=4/100) of verbal orders included the unit. If including units as a component of completeness, the number of complete verbal orders is much fewer. Only 1.0% (n=1/100) of verbal orders included the name, dose, unit of dose, and route.

Regarding verbal orders for blood, 14.3% (n=3/21) included the blood product and 33.3% (n=7/21) included the blood type. Over half (52.4%; n=11/21) of orders for blood omitted both blood product and blood type (eg, “Hang a unit of blood.”) No verbal orders for blood were complete (0%; n=0/21). Of note, 28.6% (n=6/21) of blood verbal orders were given during resuscitation of a female patient, and only 50% (n=3/6) of those orders included the blood type.

### Closed-loop Communication

Closed-loop communication was used for 73.5% (n=111/151) of orders for medication and in 61.9% (n=13/21) of orders for blood.

## DISCUSSION

Verbal orders for both medications and blood products were not universally audible or complete, and closed-loop communication was inconsistently used. This lack of audible, complete verbal orders and closed-loop communication creates room for error. In fact, this study was inspired by a patient safety event in which an incomplete verbal order for blood resulted in a young adult female trauma patient with an O− blood type receiving O+ blood during her trauma resuscitation. This case exemplifies the impact that incomplete verbal orders can have on critically ill trauma patients during their initial resuscitation. None of the care team members observed participated in any education intervention following this sentinel event. No further high-harm safety events occurred during trauma resuscitations within the data collection period, and no root cause was determined to be related to verbal ordering practices.

### Order Audibility

Orders for medications and for blood products were inaudible, a finding consistent with prior research. El-Shafy et al found 97% of orders to be audible and 26% to be closed loop.^3^ While these audibility results differ from the 78% reported in this adult ED study, the El-Shafy study was conducted in a pediatric setting, which may have impacted the ambient noise of concurrent trauma and medical resuscitations, team dynamics, and other factors. It is unclear where the enrolling study member stood in that study, which may have impacted results as well. A study by Bergs et al reported that 56% of observed orders were audible.^4^ This study was done at an adult Level 1 trauma center, but data were collected via video recording. This data collection methodology may have impacted audibility of orders and resulted in a somewhat lower percentage of audible orders recorded as compared to this study. A study team member collecting data standing among the resuscitation team most closely approximates the experience and, therefore, the audibility of orders to team members.

The environment of the ED resuscitation bay can be loud and crowded. Thus, providing audible verbal orders can be challenging for trauma captains. Nonetheless, audible verbal orders are key to patient safety. With this demonstrated room for improvement, mitigation strategies are needed and may include environmental noise control, empowering care team members to clarify indiscernible orders, and educational simulation for physicians to recognize the audibility of their orders.

### Order Content

Complete verbal orders were inconsistently given. In fact, not providing a complete verbal order was the most frequently observed deviation from best practice of all the observed measures in this study. Only 14.1% of orders for medication were complete, and no orders for blood were complete.

### Orders for Medications

Most often, a medication verbal order was incomplete because it did not include the route. In fact, 50.7% (n = 71/140) of orders for medication were incomplete due to the absence of route. Trauma captains may have omitted route as it is frequently assumed that patients were to take nothing by mouth and that medications are given intravenously (IV). If medication route were not required for order completeness, medication verbal order completeness would increase from 14.1% (n = 23/163) to 57.7% (n = 94/163). This demonstrates persistent room for improvement. Dose was absent in 38% of verbal orders. For example, a trauma captain may say, “Give some IV fentanyl.” Further, 26.4% of orders lacked even the name of the medication. For example, a trauma captain may say “Give another 50 mcg” rather than “give 50 mcg of IV fentanyl.” This certainly introduces the potential for medication error, making future QI interventions necessary. Trauma captains may deliver incomplete medication verbal orders due to relative lack of experience leading resuscitations, due to a medical knowledge deficit, or a host of other reasons. More research is necessary to evaluate the contributing factors to incomplete verbal order delivery

### Orders for Blood Products

As previously defined, a complete blood verbal order includes the blood product and blood type. Despite the fact that multiple blood products and blood types are used in our ED, no observed blood orders were complete. The importance of this is highlighted when considering blood administration in patients who may be pregnant. Due to Rh incompatibility, incorrect blood administration may contribute to future patient and fetal harm.^11^–^13^ Therefore, complete blood verbal orders are of great importance to patient safety. Given the total lack of complete blood verbal orders reflected in the data, and the high impact these orders have on patient safety, QI and safety improvement strategies are necessary.

### Closed-Loop Communication

One benefit of closed-loop communication is to identify a potential error before it occurs. This is especially important considering the frequency with which verbal orders were inaudible or incomplete. Therefore, the key to effective closed-loop communication is stating the medication or blood product that is being administered prior to administration. Throughout observation of trauma resuscitations, it was noted that closed-loop communication was not always used correctly. Upon recognizing that closed-loop communication was often used after administration of a medication or blood product, the timing of closed-loop communication was recorded as before or after administration. We assessed 60 medication and blood verbal orders for timing of closed-loop communication. Of these, 56.7% (n = 34/60) of closed-loop communication occurred after the medication or blood was already administered. Using closed-loop communication in this way limits error prevention. Further, in an ideal situation an ED nurse would use closed-loop communication both before completion of the order to ensure they heard the correct order details, and after the completion to confirm they completed the order. This practice was not observed during our data collection period. Regardless of timing, there is significant room for increased use of this best practice.

El-Shafy et al reported that 26% of orders included closed-loop communication.^3^ This represents notably fewer instances of closed-loop communication than were observed in our study. This is likely due to the definitions used to rate closed-loop communication as present. El-Shafy defined closed-loop as “audible, directed to a team member, check-back by the team member, and acknowledgment by team leader.”^3^ For this study, closed-loop communication was recorded as present if the ED nurse completing the order repeated the order back. As discussed above, it was acknowledged that the presence of closed-loop communication is likely lower than our reported 73% if only optimal closed-loop communication was recorded rather than any closed-loop communication.

Data from this study demonstrate that there is a significant quality gap between optimal verbal ordering practices and the verbal ordering practices that were observed. Therefore, opportunity exists for quality improvement. Further research is needed to determine why verbal orders are often inaudible or incomplete. Possible etiologies may include medical knowledge deficit (eg, a dose was not included in the verbal order because the resident physician did not know the correct dose), environmental factors (eg, orders were inaudible secondary to environmental noise), or the relative inexperience of the trauma captain compared to an attending physician. This study suggests that efforts should be multidisciplinary, as physicians in multiple residency training programs and nursing care team members all demonstrated ideal verbal ordering practices inconsistently. One such improvement effort may include in-situ multidisciplinary trauma simulation, which has been implemented at our institution following the data collection period of this study. Simulation has been effective in similar situations such as cardiopulmonary resuscitation using Advanced Cardiac Life Support and should be evaluated for effectiveness in trauma resuscitation improvement efforts as well.^16^ Further research is also necessary in similar situations where emergencies exist such as in medical codes and in clinical practice environments where verbal orders are used such as in operating rooms.

Of note, little has been published on recommended order content during emergent resuscitations. While best practice recommendations exist regarding the use of verbal orders, these primarily state that a physician or other healthcare professional may use a verbal order when necessary to avoid significant delay in emergent care.^14^ They do not specify order-content recommendations specific to the unique practice environment of an emergent resuscitation of a critically injured patient. Verbal orders may occur with increased frequency during emergent resuscitations; however, many recommendations for order content do not apply in this setting. Further expert opinion and society guidelines are necessary to standardize best practices for verbal ordering during trauma resuscitations and critical medical resuscitations.

## LIMITATIONS

Given the observational nature of this study in an environment that can be busy and loud, it is possible that the audibility of verbal orders is under-reported. Likewise, it is possible that certain component parts of an order were present but not heard by the study team member collecting data. However, the team member collecting data was physically located right next to the recording trauma nurse, within five feet of the trauma captain. Therefore, any inability to hear orders would mirror that of the clinical care team. In this way, data accurately reflect the communication experienced by the care team. The number of patients and staff varied throughout the observation period, thus impacting audibility. However, variability in the number of patients and staff is a clinical challenge in all emergency care settings.

We used a convenience sample of verbal orders. Many more orders were recorded for medications, during trauma alerts, and during the care of male patients. No comparative statistics were used. Nonetheless, data demonstrated the lack of audible and complete verbal orders with closed-loop communication regardless of trauma acuity, the residency training program of the captain, or the sex of the patient.

Because this was a non-blinded observational study, one might question the impact of the Hawthorne effect on verbal order practices. However, the study was not broadly discussed or announced among clinical teams. Furthermore, data were collected by a non-ATLS trained medical student who was unfamiliar to the clinical team.

This study was conducted at an academic medical center and most of the enrolled verbal orders were given by residents. Therefore, the ordering physician was most often still acquiring the skills necessary to expertly resuscitate trauma patients. Verbal-order audibility and completeness may be present more frequently in a non-academic setting, where ordering physicians have completed residency training. While this limits the generalizability of our data to settings where attending physician-only teams resuscitate trauma patients, many tertiary care and Level 1 trauma centers are at academic medical centers, and the data are, therefore, applicable to many EDs in which trauma resuscitations are performed.

## CONCLUSION

Audible, complete verbal orders and closed-loop communication are underused during the multidisciplinary resuscitation of trauma patients in our ED. Findings were consistent regardless of the severity of trauma activation or the training program of the trauma captain. This represents a multidisciplinary QI opportunity wherever verbal orders are used. Future research is needed in other clinical practice environments where verbal orders are used, and after QI initiatives have been implemented.

## Figures and Tables

**Table 1:** Trauma activation criteria and resources; trauma alerts are the highest level of activation in which the full team will respond.

Trauma Activation Criteria	Trauma Activation Resources
Trauma Alert Glasgow Coma Score (GCS) < 9 Systolic blood pressure (SBP) < 90 at any time Heart rate (HR) < 50 or > 130 Respiratory rate < 10 or > 29 breaths/minute Intubated patients transported from the scene or transferred from another facility Patients with respiratory compromise or obstruction Transfer patients from other hospitals who require blood to maintain vital signs Gunshot wound to the head, neck, chest, back, or abdomen Gunshot wound to extremities with active bleeding or non-palpable pulses Tourniquet on any extremity or wound-packing with continuous pressure Transfer with known head bleed and GCS ≤ 13 Transfer with known multisystem trauma (≥ 2 systems) Geriatric criteria (> 65 years old): SBP < 110 at any time, and HR > 100 at any time Hypothermia from immersion or suspected exposure with vital sign criteria as above Clinician discretion	The trauma alert team will respond immediately to the trauma resuscitation bays to participate in resuscitation and therapeutic decision-making: Trauma Surgery Faculty – within 15 minutes of patient arrival Trauma Surgery Senior Resident Emergency Medicine (EM) Faculty EM Residents Anesthesiology Emergency Department (ED) Nurses (Recorder, Left, Right) ED Technician Respiratory Therapy Radiology Technician Social Services Pharmacy
Trauma Call (mid-acuity) GCS 9–12 with mechanism attributed to trauma Stab wound to the head, neck, chest, back, or abdomen Any penetrating injury to the back Penetrating injury proximal to the elbow or knee Flail chest Combination trauma and burns ≥ 2 proximal long bone fractures Pelvic fracture Open or depressed skull fracture Transfer with known head bleed and GCS > 13 New-onset paralysis Amputation above the wrist or ankle. Rigid or distended abdomen related to trauma mechanism Geriatric criteria (> 65 years old): Any fall greater than standing height Motor vehicle collision > 25 mph Pedestrian struck Falls > 20 feet High-risk auto collision: Intrusion, including roof: > 12 inches occupant site, or > 18 inches any site Ejection (partial or complete) Death in same passenger compartment Vehicle telemetry data consistent with high risk of injury Pedestrian/bicyclist/motorcycle/recreational vehicle thrown, run over, or with significant impact (> 20 mph) Injured, pregnant trauma patient > 22 weeks Hanging – only with patient injury Inter-hospital transfer of trauma (unless direct admission is arranged) Clinician discretion	The trauma call team will respond immediately to the trauma resuscitation bays to participate in resuscitation and therapeutic decision-making. EM Faculty Trauma Surgery Senior Resident EM Residents ED Nurses (recorder, left, right) ED Technician Respiratory Therapy Social Services Trauma Surgery Faculty – within 6 hours of patient arrival

**Table 2:** Characteristics of verbal orders, reported as absolute number and percentage.

Characteristics	Number of verbal orders (N = 186)
Trauma activation	
Call	72 (38.7%)
Alert	114 (61.3%)
Role of captain	
EM resident	138 (74.2%)
Surgery resident	34 (18.3%)
Other physician	14 (7.5%)
Patient sex	
Female	54 (29.0%)
Male	132 (71.0%)
Medication orders (n = 163)	
Audible	127 (77.9%)
Name	120 (73.6%)
Dose	101 (62.0%)
Route	29 (17.8%)
Closed-loop communication	111 (73.5%)
Complete	23 (14.1%)
Blood orders (n = 21)	
Audible	16 (76.2%)
Blood product	3 (14.3%)
Blood type	7 (33.3%)
Closed-loop communication	13 (61.9%)
Complete	0 (0%)

More trauma alerts than trauma calls were included. Emergency medicine residents gave most of the verbal orders, and male patients outnumbered female patients. There was < 100% compliance with verbal order audibility, completeness, and closed-loop communication.
